# Cortical laminar necrosis in dengue encephalitis—a case report

**DOI:** 10.1186/s12883-017-0855-9

**Published:** 2017-04-20

**Authors:** Ravindra Kumar Garg, Imran Rizvi, Rajan Ingole, Amita Jain, Hardeep Singh Malhotra, Neeraj Kumar, Dhruv Batra

**Affiliations:** 1 Department of Neurology, King George’s Medical University, UP, Lucknow, 226003 India; 2 Department of Microbiology, King George’s Medical University, UP, Lucknow, 226003 India

**Keywords:** Cortical laminar necrosis, Encephalitis, Encephalopathy, Magnetic resonance imaging, Dengue virus, Case report

## Abstract

**Background:**

Dengue encephalitis is a rare neurological manifestation of dengue fever. Its clinical presentation is similar to other viral encephalitides and encephalopathy. No single specific finding on magnetic resonance imaging of dengue encephalitis has yet been documented. They are highly variable and atypical.

**Case presentation:**

A 15-year boy presented with fever, the headache and altered sensorium of 12-day duration. On neurological examination, his Glasgow Coma Scale score was 10 (E3M4V3). There was no focal neurological deficit. Laboratory evaluation revealed leukopenia and marked thrombocytopenia. Dengue virus IgM antibody was positive both in serum and cerebrospinal fluid. Magnetic resonance imaging of the brain revealed signal changes in bilateral parietooccipital and left frontal regions (left hemisphere more involved than the right hemisphere). There was gyriform enhancement bilateral parietooccipital regions consistent with cortical laminar necrosis. Bilaterally diffuse subcortical white matter was also involved and subtle T2 hyperintensity involving both basal ganglia was noted. Gradient echo sequence revealed presence of hemorrhage in the subcortical white matter. Patient was treated conservatively and received platelet transfusion. Patient became fully conscious after 7 days.

**Conclusion:**

In a patient with highly suggestive dengue e\ephalitis, we describe an unusual magnetic resonance imaging finding. This report is possibly the first instance of cortical laminar necrosis in such a setting.

## Background

Currently, dengue infection is endemic in more than 100 countries. The incidence of dengue has increased dramatically in recent decades. World Health Organization, currently, estimates that 390 million dengue infections occur per year, of which 96 million manifest clinically; about 2.5% of those affected dies. Dengue virus is a single-stranded RNA virus belonging to the *Flaviviridae* family. There are four serotypes of dengue virus (DENV-1 to DENV-4), of which serotypes DENV-2 and DENV-3 have been implicated most frequently in dengue fever. The involvement of the central nervous system has been described with all 3 classical types of dengue, dengue fever, dengue hemorrhagic fever, and dengue shock syndrome [1].

Encephalitis has been reported worldwide as a severe complication in patients infected by dengue virus. Dengue virus encephalitis often presents as acute encephalitis syndrome during dengue epidemics and can be seen in all 3 classical types of dengue. Indirect mechanisms such as impaired kidney and liver dysfunctions, hypoxic-ischemic insults, and other metabolic disorders can affect brain function and clinically present as encephalopathy. In addition to direct viral involvement of brain, there are several other pathologies like ischemic and hemorrhagic strokes, subdural hematoma, cerebral venous thrombosis, and acute disseminated encephalomyelitis that can affect the brain [2–6] (Table 1).

**Table 1:** Neuroimaging spectrum of dengue-associated encephalitis/encephalopathy

Clinical syndrome	Neuroimaging features	Pathogenesis
Diffuse brain involvement		
Encephalopathy	Often normal	Impaired liver and renal dysfunction Ischemic-hypoxemic injury
Acute encephalitis	Usually normal; diffuse cortical and periventricular, hyperintensities	Direct viral invasion of brain
Acute hemorrhagic encephalitis	GRE sequence shows areas of blooming indicating hemorrhage	Direct viral invasion of brain
Acute disseminated encephalomyelitis	Periventricular white matter lesions	Post-infectious demyelination
Vascular complications		
Cerebral venous thrombosis	Thrombosis of venous system of brain	Severe dehydration and hypotension
Intracerebral hemorrhage	Cerebral parenchymal, subdural or subarachnoidal hematoma	Thrombocytopenia, disseminated intravascular coagulation and associated coagulopathies
Ischemic stroke	Multiple infarctions	Vasculitis, infective thromboembolism
Focal encephalitis		
Cerebellitis Rhombencephalitis	Hyperintensities in brainstem and cerebellum	Direct viral invasion

In most cases with dengue virus encephalitis, neuroimaging is normal [7]. Whenever, neuroimaging is abnormal, changes are usually non-specific in terms of etiology but may provide a hint towards the possible pathophysiological process. We report a case highly suggestive of dengue encephalitis with an unusual magnetic resonance imaging finding in brain.

## Case presentation

**Fig. 1:**
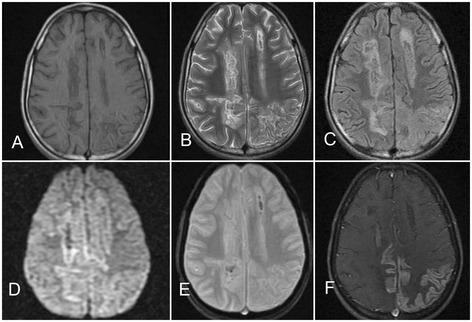
Axial sections of MRI of the brain depict curvilinear hyperintense cortical lesions in bilateral parietal and occipital areas on T1 W (**a**), T2 W (**b**) and T2 fluid attenuated inversion recovery (**c**) sequences. Mild gyral diffusion restriction is noted on diffusion weighted sequence (**d**) while blooming is seen on gradient echo sequence (**e**). Post-contrast T1 W sequence (**f**) depicts gyral enhancement

A 15-year-old boy presented to our University Hospital, a tertiary care neurology facility, with fever, headache, and altered sensorium of 12-day duration. At admission, he was febrile (101 °F), with a pulse rate of 110/min and a blood pressure recording of 114/74 mmHg; his Glasgow Coma Scale score was 10 (E3M4V3). There were no petechiae or signs of bleeding over the skin or any mucosal surface. There was no focal neurological deficit and neurological examination was normal, including (absence of) signs of meningeal irritation. Laboratory evaluation revealed the following estimations: hemoglobin of 11 g/dl, total leukocyte count of 3300 cells/mm^3^, and platelet count of 22,000 cells/mm^3^ which dropped to 8000 cells/mm^3^ on next day. His aspartate aminotransferase level was 155 U/L, alanine aminotransferase was 140 U/L and alkaline phosphatase was 56 U/L. Additional biochemical parameters, renal function tests, blood sugar, electrolytes, and arterial blood gas analysis, were normal. Malarial parasite was not detected in the peripheral blood smear. The cerebrospinal fluid examination, cytological and biochemical, was normal. IgM antibody against dengue virus was positive both in serum and cerebrospinal fluid; meanwhile, dengue NS1 antigen was negative. Electroencephalography revealed generalized slowing. On magnetic resonance imaging of the brain, signal changes were seen in bilateral parietooccipital and left frontal region (left hemisphere was more involved than the right hemisphere). There were diffuse subcortical white matter changes along with suggestion of hemorrhage on gradient echo sequence. Subtle hyperintensity on T2 W images was also noted in bilateral basal ganglia. Gadolinium-contrast study revealed a gyriform enhancement suggestive of cortical laminar necrosis (Fig. 1). He was managed conservatively and given platelet transfusion. The patient responded well to management and became fully conscious in 7 days.

## Discussion

Cortical laminar necrosis is an imaging term classically used to describe high-intensity cortical lesions in a gyriform distribution on T1-weighted magnetic resonance imaging. Cortical laminar necrosis is, pathologically, characterized as necrosis of particular cortical laminae involving neurons, glial cells, and cerebral blood vessels. Cortical laminar necrosis is predominantly caused by hypoxia and metabolic disorders, like hypoglycemia, intoxication, hypoxic-ischemic encephalopathy, renal and hepatic dysfunction, but may also be seen in patients with encephalitis [8–10]. We describe the occurrence of cortical laminar necrosis in a setting highly suggestive of dengue infection, possibly the first instance, in a 15-year-old boy.

Dengue virus was, at large, considered a non-neurotropic virus. With an increase in awareness and improvement in laboratory techniques, dengue virus or dengue viral components are being isolated from brain tissue or from cerebrospinal fluid specimens [11–13]. The mechanism of dengue virus invasion of the central nervous system, and its consequences, is not completely understood. It is supposed that the neurological complications of dengue virus are caused by many mechanisms, singularly or in a combination, like systemic metabolic abnormalities, liver and kidney dysfunctions, direct infection, post-infectious immune disorders, abnormal vascular permeability and flow abnormalities, and coagulopathies. Possibly, the virus enters the central nervous system via infected macrophages. In an experimental study, Bordignon and co-workers noted that a mutant dengue virus-1 escaped immune defense mechanism of the body and caused a severe meningo-encephalitis in mice [14]. In another study that included 84 patients with fatal dengue meningoencephalitis, dengue virus serology in cerebrospinal fluid specimens was positive in 50% of the patients. These findings indicate that dengue virus has neurotropic properties and actively invades central nervous system [11]. Dengue virus-associated cerebrospinal fluid pleocytosis, cerebral parenchymal inflammatory cell infiltrates, and intrathecal synthesis of dengue-specific antibodies also indicate active viral invasion of brain [15].

A wide variety of neuroimaging changes have been described in dengue encephalitis. Not all patients have neuroimaging abnormalities. Bhoi and co-workers, in a series of 20 patients, noted neuroimaging abnormalities in 50% of patients with dengue virus infection [16]. Multifocal hyperintensities in bilateral periventricular zones, including basal ganglia, may be seen on T2 W and fluid attenuated inversion recovery sequences [3]. Brainstem, cerebellum, corpus callosum and thalamus may also be involved [17]. In many patients with acute hemorrhagic encephalitis, gradient echo sequences reveal patchy areas of blooming suggestive of hemorrhage [18]. In many patients with dengue infection, encephalopathy is being attributed to acute disseminated encephalomyelitis. In such cases, T2-weighted magnetic resonance imaging reveals signal abnormalities in the subcortical and periventricular white matter, with or without gray matter involvement. Dengue-associated acute disseminated encephalomyelitis, being an immune mediated disorder of the central nervous system, has been shown to respond well to intravenous methylprednisolone [2].

In our case, we kept the possibility of direct dengue virus infection as the foremost cause of cortical laminar necrosis. Metabolic abnormalities, as depicted by *mildly* deranged liver functions, was kept as the next possibility. As the patient was hemodynamically stable, hypoxic-ischemic insult seems unlikely and the spatial distribution of lesions did not favor the occurrence of posterior leukoencephalopathy. Although evidence of blooming is known in patients with encephalitis, thrombocytopenia in the present scenario might have had a contributory role in the characteristic radiological presentation.

Diagnosis of dengue encephalitis is often not difficult in the setting of a dengue epidemic and a patient presenting with febrile encephalopathy and demonstration of anti-dengue IgM antibodies or dengue genomic material in serum and/or cerebrospinal fluid could be diagnosed as dengue encephalitis [19]. However, in low incidence areas and non-endemic zones, a high index of suspicion should be kept and an effort should be made to rule out other causes of viral encephalitis prevalent in that area [20]. Dengue NS-1 antigen might not be demonstrable in all cases especially those who present after 1 week of onset of symptoms. A low platelet count and altered liver and renal function may help in making diagnosis of dengue encephalitis/encephalopathy.

## Conclusion

There is no characteristic neuroimaging finding in dengue encephalitis. Frequently, changes in white matter and deep gray matter have been described. We describe an unusual magnetic resonance imaging finding in a patient dengue encephalitis. Cortical laminar necrosis has never been described in dengue encephalitis.

## Abbreviations

DENV: Dengue virus
RNA: Ribonucleic acid
